# Effects of co-cultiv*ation* with *Gracilariopsis lemaneiformis* on the explosive growth and photosynthesis of *Ulva prolifera*

**DOI:** 10.1371/journal.pone.0344202

**Published:** 2026-03-10

**Authors:** Wei Zhou, Wentao Lu, Yi Hao, Yuyue Zhou, Yuru Yan, Qun Liu, Jiatao Xu, Juntian Xu, Chen Liang, Xincheng Song, Jiahu Xia, Jinguo Wang

**Affiliations:** 1 Key Laboratory of Coastal Salt Marsh Ecosystems and Resources Ministry of Natural Resources, Jiangsu Ocean University, Lianyungang, China; 2 Marine Geological Survey of Jiangsu Province, Nanjing, China; 3 Lianyungang Ocean Development Co., Ltd., Lianyungang, China; 4 Lianyungang Marine and Fishery Development Promotion Center, Lianyungang, China; 5 Lianyungang Shengyang Aquatic Seed Breeding Co., Ltd., Lianyungang, China; King Abdulaziz University, SAUDI ARABIA

## Abstract

The recurring outbreak of *Ulva prolifera* green tides in the Yellow Sea poses a serious threat to coastal ecosystems and coastal-related industries, necessitating the development of environmentally friendly control strategies. In this study, in situ experiments were conducted using two light intensity levels and five co-cultivation biomass density combinations to investigate their effects on the growth and photosynthetic physiology of *Gracilariopsis lemaneiformis* and *U. prolifera*. The results demonstrated a significant interactive effect of light intensity and biomass density on the competitive outcome between the two species. Specifically, the relative growth rate of *U. prolifera* significantly decreased with increasing biomass density of *G. lemaneiformis–*an inhibitory effect that was particularly pronounced under high light intensity. High light enhanced the competitive suppression capacity of *G. lemaneiformis*, likely associated with shading effect due to its growth, while reduced photosystem stability of *U. prolifera* under high light further exacerbated the inhibition. Under low light, *U. prolifera* alleviated competitive pressure by increasing its chlorophyll content. *G. lemaneiformis* suppressed *U. prolifera* through resource competition, and a biomass density ratio of 1:4–1:6 (*U. prolifera*: *G. lemaneiformis*) was found to balance effective ecological control with sustained growth of *G. lemaneiformis*. The research findings provide novel scientific insights and empirical data to support the advancement of *G. lemaneiformis* aquaculture and the development of biological control strategies for mitigating green tides.

## Introduction

The recurrent outbreaks of large-scale green tides has become a serious challenge for the global coastal ecosystems. Since 2007, the Yellow Sea in China has experienced annual outbreaks of green tides, predominantly dominated by *Ulva prolifera* [1,2]. These outbreaks not only degrade coastal landscapes and obstruct shipping channels, but also pose severe threats to coastal fisheries, aquaculture, and tourism [3,4]. The complex life history and multiple reproductive modes of *U. prolifera* are important reasons for its dominance in the Yellow Sea green tide region. Additionally, environmental changes such as global warming and coastal eutrophication are significant potential drivers of green tide outbreaks [5]. It is noteworthy that the formation of *U. prolifera* green tides is a complex ecological process resulting from the synergistic interaction between biological characteristics and environmental factors [4,6]. Features such as their extensive spatial distribution, excessive biomass accumulation, and rapid diffusion make it difficult for traditional single-approach control methods to achieve long-term management. This practical challenge places higher demands on the scientific rigor, systematic approach, and innovation required for green tide prevention and control efforts.

Current mitigation strategies for green tides formed by *U. prolifera* fall into three categories: physical, chemical, and biological methods [7]. Physical methods utilize satellite remote sensing to track the drift path of green tides in real-time, assisting salvage vessels in targeted removal of *U. prolifera*. These methods are costly and consume human and material resources [1]. Traditional chemical methods involve the application of compounds such as strong oxidants, heavy metal compounds, and acid treatments, which inevitably cause environmental pollution and other problems [8]. In contrast, biological control methods have garnered growing attention due to their environmentally friendly and potentially sustainable characteristics. However, their mechanisms of action, application conditions, and large-scale feasibility still require in-depth investigation [9]. Therefore, based on elucidating the ecological mechanisms, developing new biological control approaches grounded in biological interactions is of significant importance for enriching green-tide management strategies.

In China, *G. lemaneiformis* has become the second-largest cultivated seaweed. It not only holds significant aquacultural economic value but also demonstrates notable potential for nutrient absorption and ecological remediation [10,11]. Studies conducted under laboratory conditions have confirmed that economic macroalgae can not only inhibit *U. prolifera* growth by competing for light and nutrients, but also interfere with its photosynthesis and growth through the release of allelochemicals [12–14]. The competitive growth among macroalgae is influenced by multiple environmental factors such as light, nutrients, temperature, and water flow. Especially in complex nearshore ecosystems, changes in a single environmental variable may alter interspecific competition patterns through niche reconstruction. Therefore, it is necessary to analyze the interactive effects of multiple factors under conditions that closely resemble natural environments.

Light intensity and biomass density are key environmental factors regulating the competition among macroalgae [15]. As an opportunistic species, the growth of *U. prolifera* is highly dependent on light and nutrient availability [16,17]. In contrast, *G. lemaneiformis* exhibits broader adaptability to light conditions [18]. The outcome of competition between the two species may vary with changes in light conditions and relative biomass [19,20]. However, most existing studies have focused on single-factor experiments or laboratory simulations, resulting in a lack of systematic understanding of how light and biomass density interact to regulate the competitive dynamics and underlying physiological mechanisms of these two algae under in situ conditions. Especially in field environments, clarifying the inhibitory effect of *G. lemaneiformis* on the competitive growth of *U. prolifera* holds significant practical value for optimizing cultivation strategies of *G. lemaneiformis* and advancing biological control technologies for green tides.

In this study, an in situ experiment was conducted with two light gradients and five co-cultivation biomass densities to systematically investigate the effects of different light intensities and biomass densities on the growth rates, photosynthetic activity, and biochemical parameters of *G. lemaneiformis* and *U. prolifera*. The objective of this study was to elucidate the interspecific competitive dynamics among macroalgae and provide a scientific basis for optimizing *G. lemaneiformis* cultivation models and innovating biological control strategies for green tides.

## Materials and methods

### Experimental materials

*G. lemaneiformis* used in the experiment was collected from aquaculture rafts in Luoyuan Bay, Fujian Province (26°25'23.01" N, 119°41'32.12" E). *Ulva prolifera* was collected from Zaihaiyifang Park in Lianyungang City, Jiangsu Province (34°36'06" N, 119°46'12" E). The collected thalli of *G. lemaneiformis* and *U. prolifera* were placed in a portable temperature-controlled box at 4 ℃ and transported to the laboratory. Thalli were cleaned with filtered and sterilized seawater to remove epiphytes and other contaminants. Healthy thalli were selected for pre-cultivation in a smart illumination incubator (GXZ-500C, China) at a temperature of 20 ℃, a light intensity of 100 μmol photons m ⁻ ² s ⁻ ¹, and a photoperiod of 12 h light: 12 h dark, before being used in subsequent experiments.

### Experimental design

The experiment investigated two environmental factors: light intensity and biomass density. Considering the optimal temperature and light intensity for the growth of *G. lemaneiformis* and *U. prolifera*, a two-week in situ experiment was conducted in April in the intertidal zone near Dongbawei, Guanyun County, Lianyungang City, Jiangsu Province, China (34°29'00.04" N, 119°46'02.23" E). Two light intensity levels were set: Low Light (LL: covered with 3 layers of shading net) and High Light (HL: covered with 2 layers of shading net). Under each light level, five growth biomass density gradients were established: *Ulva prolifera* monoculture (UP); *Gracilariopsis lemaneiformis* monoculture (GL); *U. prolifera*: *G. lemaneiformis* = 1:2 (1U2G); *U. prolifera*: *G. lemaneiformis* = 1:4 (1U4G); and *U. prolifera*: *G. lemaneiformis* = 1:6 (1U6G). The biomass density ratios were set based on chlorophyll *a* content, with the unit density defined as 0.5 g for *U. prolifera* and 0.8 g for *G. lemaneiformis*. UP and GL represent the monoculture treatment groups, designated as IC (individual culture). This resulted in 10 treatment groups combining light intensity and culture biomass density (LL: UP, GL, 1U2G, 1U4G, 1U6G; HL: UP, GL, 1U2G, 1U4G, 1U6G), with three replicates per combination. All experimental algae were cultured in 25 L tanks placed on a coastal embankment slope, with each tank filled with 20 L of natural seawater collected on-site. Additionally, fresh seawater was replaced every two days to meet the growth requirements of the thalli and to avoid nutrient limitation. During the experimental period, the air temperature ranged from 12.15 to 28.96 ℃; water temperature ranged from 13.77 to 23.77 ℃. The pH varied between 8.12 and 8.26, and salinity ranged from 31.2 to 32.5. The concentrations of dissolved inorganic nitrogen (DIN) and dissolved inorganic phosphorus (DIP) were measured both at the start of the experiment and at each water exchange. Initial concentrations: DIN = 29.96 ± 1.34 µmol L^-1^, DIP = 1.16 ± 0.05 µmol L^-1^; concentrations at water exchange: DIN = 29.28 ± 2.24 µmol L^-1^, DIP = 1.12 ± 0.09 µmol L^-1^.

### Environmental solar radiation measurement

A solar radiation receiver (LoggerNet CR3000, Tiannuo, Beijing, China) was used to monitor and record solar radiation (PAR, μmol photons m ⁻ ² s ⁻ ¹) in real-time every minute during the experimental period. Daily dose was calculated in kJ m ⁻ ².

### Growth measurement

Fresh weight of algae was measured every two days. During measurement, the same number of layers of absorbent paper was used to gently press the thallus surface to remove water, maintaining consistent pressing time. All fresh weight of algae measurements were performed by the same operator using the same balance to minimize human and instrumental errors. Relative Growth Rate (RGR, % d ⁻ ¹) was calculated as follows:


$$\begin{array}{c} RGR\;(\%\;day^{\text{-1}})=ln\;(W_{t}/W_{0})/t\times 100 \end{array}$$

Where: *Wₜ* is the fresh weight (g) of algae on day t, *W₀* is the initial fresh weight (g), and *t* is the cultivation time in days (d).

### Respiration rate measurement and net photosynthetic rate

Respiration rate (R_d_) and net photosynthetic rate (P_n_) of *G. lemaneiformis* and *U. prolifera* were measured using an oxygen electrode (YSI-5300A, YSI Inc., USA). Approximately 0.1 g of algae was cut into 1 cm segments and dark-adapted at 20 ℃ for 12 hours to minimize errors from mechanical damage. The instrument was zeroed by adding 8 ml of pure water to the reaction chamber while continuously purging with N_2_, adjusting the reading to zero. The solution was then repeatedly pipetted for 20 minutes, and the instrument reading was adjusted to 100. The temperature was controlled at 20 ℃ using a constant temperature circulator. Algae segments were placed in the reaction chamber for measurement; values were recorded once stable, at one-minute intervals. To measure respiration rate, a light-tight dark box was placed over the reaction chamber to create complete darkness, and the change in O_2_ concentration was recorded. To measure net photosynthetic rate, the distance between the halogen lamp and the reaction chamber was adjusted to simulate outdoor light conditions, and the change in O_2_ concentration was recorded.

### Chlorophyll fluorescence parameter measurement

Chlorophyll fluorescence parameters of *G. lemaneiformis* and *U. prolifera* were measured using a handheld pulse amplitude modulation chlorophyll fluorometer (Aquapen AP 100, Photon Systems Instruments, Drásov, Czech Republic). The effective quantum yield of photosystem II (Yield) was measured every 1.5 hours from 8:30 AM to 5:30 PM for algae in each treatment group. Relative Electron Transport Rate (rETR) was determined at eight actinic light intensities [0, 10, 20, 50, 100, 200, 500, and 1000 μmol photons m ⁻ ² s ⁻ ¹] [21]:


rETR=Yield×0.5×PAR

Where Yield is the effective quantum yield; 0.5 is the ratio of absorbed to total incident light energy; PAR is the light intensity.

Rapid Light Curves (RLC) were fitted according to the following formula [22]:


$$rETR=PAR/(a\times PAR^{\text{2}}+b\times PAR+c)$$

Where rETR is the relative electron transport rate, PAR is the light intensity, and a, b, c are fitting parameters.

Maximum relative electron transport rate (rETRₘₐₓ), photosynthetic efficiency (α), and saturating irradiance (Iₖ) were calculated from *a*, *b*, *c*:


$$rETR_{max}=1/[b+2\times (a\times c{)}^{1/2}]$$

α=1/c

$$Ik=rETR_{max}/\alpha$$

### Photosynthetic pigment content determination

Approximately 0.05 g of *G. lemaneiformis* or *U. prolifera* thalli was cut into small pieces, placed in a centrifuge tube with 5 ml of anhydrous methanol, and kept in the dark at 4 ℃ for 24 hours. The supernatant was taken after shaking. Absorbance at wavelengths 470 nm, 653 nm, and 666 nm was measured using an ultraviolet spectrophotometer. Chlorophyll *a* (Chl *a*, mg g ⁻ ¹ FW) and carotenoid (Car., mg g ⁻ ¹ FW) contents were calculated as follows [23]:


$$Chl\;a=15\mathrm{.65}\times A_{666}-7\mathrm{.53}\times A_{653}$$

$$Car.=(1000\times A_{470}+1403\mathrm{.57}\times A_{666}-3473\mathrm{.87}\times A_{653})/211$$

Where Chl *a* is chlorophyll *a* content; Car. is carotenoid content (mg g ⁻ ¹ FW); *A₄₇₀* nm, *A₆₅₃* nm, *A₆₆₆* nm are absorbance values at 470 nm, 653 nm, and 666 nm, respectively.

Approximately 0.05 g of *G. lemaneiformis* thalli was fully ground in a mortar. The ground sample was transferred to a centrifuge tube containing 5 ml of phosphate buffer. The sample was centrifuged at 5000 r min⁻^1^ for 15 min at 4 ℃, and the supernatant was collected. Absorbance at wavelengths 455 nm, 564 nm, 592 nm, 618 nm, and 645 nm was measured using an ultraviolet spectrophotometer. Phycoerythrin (PE, mg g ⁻ ¹ FW) and phycocyanin (PC, mg g ⁻ ¹ FW) contents were calculated as follows [24]:


$$PE=[(A_{564}-A_{592})-(A_{455}-A_{592})\times 0\mathrm{.2}]\times 0\mathrm{.12}$$

$$PC=[(A_{618}-A_{645})-(A_{592}-A_{645})\times 0\mathrm{.51}]\times 0\mathrm{.15}$$

Where PE is phycoerythrin concentration; PC is phycocyanin concentration; *A*_*645*_ nm, *A*_*618*_ nm, *A*_*592*_ nm, A_*564*_ nm, A_*455*_ nm are absorbance values at 645 nm, 618 nm, 592 nm, 564 nm, and 455 nm, respectively.

### Soluble protein and soluble carbohydrate determination

Soluble protein (SP) content of *G. lemaneiformis* and *U. prolifera* was determined using the Bradford method [25]. Approximately 0.05 g of algae was ground in a mortar, mixed with 5 ml of phosphate buffer solution, and transferred to a centrifuge tube. The mixture was centrifuged at 5000 r min^-1^ for 15 min at 4 ℃. Then, 1 ml of supernatant was mixed with 4 ml of Coomassie Brilliant Blue solution. Absorbance was measured at 595 nm using an ultraviolet spectrophotometer. Soluble carbohydrate (SC) content was determined using the sulfuric acid-anthrone colorimetric method [26]. Approximately 0.05 g of algae was fully ground in a mortar, mixed with 5 ml of phosphate buffer solution, boiled for 1 hour, and centrifuged at 5000 r min^-1^ for 10 min. Then, 1 ml of supernatant was mixed with 4 ml of sulfuric acid-anthrone solution, placed in a boiling water bath for 10 min, and absorbance was measured at 620 nm using an ultraviolet spectrophotometer.

### Data analysis

Experimental data are expressed as “mean ± standard deviation (mean ± SD)”. Graphing and statistical analyses were performed using Origin 2021 and SPSS 26.0 software, respectively. All experimental data met the assumptions of normality (*P* > 0.05) and homogeneity of variance (*P* > 0.05). Differences among treatment groups were analyzed using one-way analysis of variance (One-way ANOVA), with Tukey’s multiple comparison test used to determine statistically significant differences between groups. Two-way analysis of variance (Two-way ANOVA) was used to compare the effects of light intensity and biomass density on the relative growth rate, net photosynthetic rate, respiration rate, chlorophyll fluorescence parameters, chlorophyll *a*, carotenoids, phycoerythrin, phycocyanin, soluble protein, and soluble carbohydrate content of *G. lemaneiformis* and *U. prolifera*. A significance level of *P* < 0.05 was used for all statistical analyses.

## Results

### Environment solar radiation

**Fig 1:**
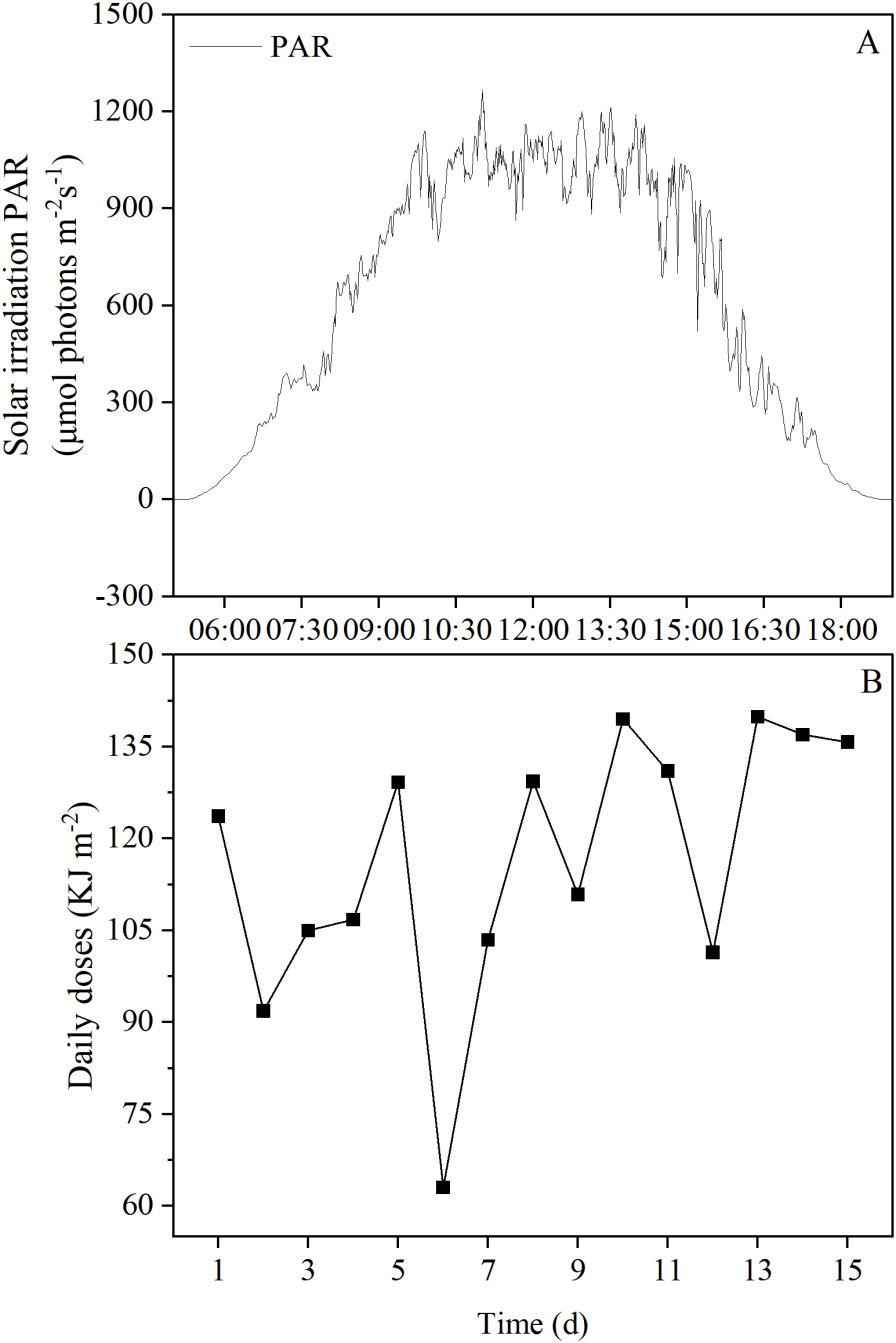
The daily average variation (A) and daily cumulative variation (B) of outdoor solar radiation over 15 days during the experiment.

The average daily variation of outdoor solar radiation during the experimental period is shown in Fig 1. The peak of daily solar radiation variation occurred at 11:01 AM, with an intensity of 1267 μmol m ⁻ ² s ⁻ ¹ (Fig 1A). The daily cumulative radiation ranged from 63.03 kJ m ⁻ ² to 139.88 kJ m ⁻ ², with a 15-day average daily cumulative radiation of 116.50 kJ m ⁻ ² (Fig 1B).

### Growth

**Fig 2:**
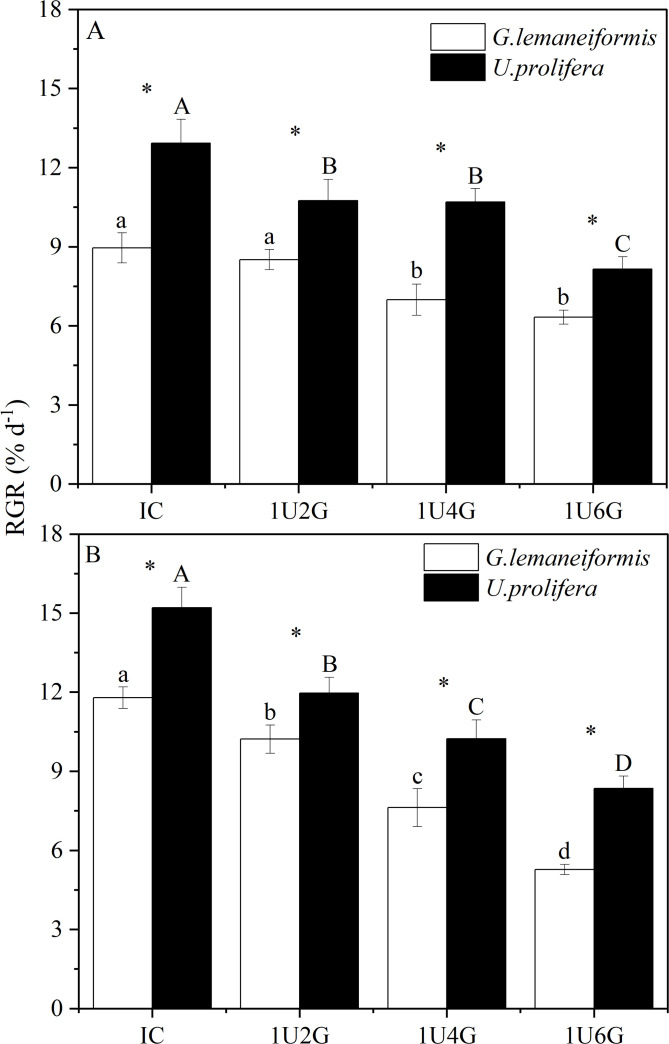
Relative Growth Rate (RGR) of *G. lemaneiformis* and *U. prolifera* under low light (LL; A) and high light (HL; B) intensities at different culture densities. IC: Individual culture (monoculture); 1U2G, 1U4G, and 1U6G: Co-culture with *G. lemaneiformis* to *U. prolifera* biomass density ratios of 1:2, 1:4, and 1:6, respectively. Different lowercase letters indicate significant differencesin RGR among *G. lemaneiformis* treatments across different culture densities. Different uppercase letters indicate significant differences in RGR among *U. prolifera* treatments across different culture densities. * indicates a significant difference in RGR between *G. lemaneiformis* and *U. prolifera* at the same culture density.

Changes in both light intensity and biomass density significantly affected the relative growth rate (RGR) of *G. lemaneiformis* and *U. prolifera* (*P* < 0.05), and their interaction also had a significant effect on the RGR of both species (*P* < 0.05) (S1 Table in S1 File). As shown in Fig 2A, under low light intensity, the relative growth rates of both *G. lemaneiformis* and *U. prolifera* were highest in monoculture, at 8.95% ± 0.57% d ⁻ ¹ and 12.92% ± 0.92% d ⁻ ¹, respectively. The growth rate of *G. lemaneiformis* in the 1U2G treatment group was not significantly different from the IC group (*P* > 0.05). *G. lemaneiformis* RGRs in the 1U4G and 1U6G groups decreased significantly by 21.90% and 29.27%, respectively, compared to the IC group. The growth rate of *U. prolifera* in the 1U2G, 1U4G, and 1U6G groups decreased significantly by 16.80%, 17.18%, and 36.92%, respectively, compared to the IC group. Under high light intensity (Fig 2B), the growth rates of both *G. lemaneiformis* and *U. prolifera* gradually decreased with increasing *G. lemaneiformis* biomass density, peaking in monoculture at 11.97% ± 0.41% d ⁻ ¹ and 15.21% ± 0.77% d ⁻ ¹, respectively. Compared to the monoculture group, the RGR of *G. lemaneiformis* in the respective co-culture groups decreased significantly by 13.32%, 35.37%, and 55.30% (*P* < 0.05), and the RGR of *U. prolifera* decreased significantly by 21.30%, 32.74%, and 45.10% (*P* < 0.05). Combining both light conditions, the relative growth rates of both algae species showed a decreasing trend with increasing *G. lemaneiformis* biomass density. Furthermore, under the same culture conditions, the RGR of *U. prolifera* was significantly higher than that of *G. lemaneiformis* (*P* < 0.05), with increases ranging from 14.62% to 36.89%.

### Respiration rate and net photosynthetic rate

**Fig 3:**
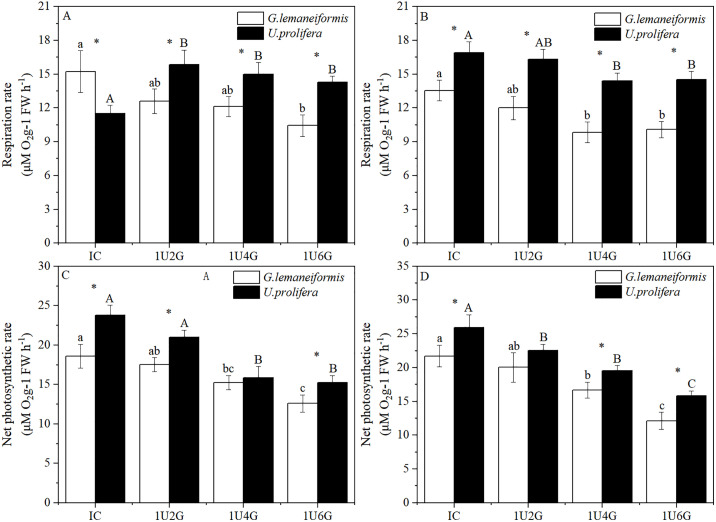
Respiratory rate (A, B) and net photosynthetic rate (C, D) of *G. lemaneiformis* and *U. prolifera* under low light (LL; A, C) and high light (HL; B, D) intensities at different culture densities. IC: Individual culture (monoculture); 1U2G, 1U4G, and 1U6G: Co-culture with *G. lemaneiformis* to *U. prolifera* biomass density ratios of 1:2, 1:4, and 1:6, respectively. Different lowercase letters indicate significant differences among *G. lemaneiformis* treatments at the same light intensity level across different culture densities. Different uppercase letters indicate significant differences among *U. prolifera* treatments at the same light intensity level across different culture densities. * indicates a significant difference between *G. lemaneiformis* and *U. prolifera* at the same culture density.

Biomass density significantly affected the dark respiration rate (R_d_) of both *G. lemaneiformis* and *U. prolifera* (*P* < 0.05), while light intensity did not have a significant effect on R_d_ (*P* > 0.05). However, the interaction between light intensity and biomass density significantly affected R_d_ for both species (*P* < 0.05) (S2 Table in S1 File). As shown in Fig 3A, under low light intensity, R_d_ of *G. lemaneiformis* was significantly different only between the IC and 1U6G groups (*P* < 0.05), with the value in the 1U6G group being 31.50% lower than in the IC group. No significant differences were observed among other groups. For *U. prolifera* under low light, the R_d_ in the IC group reached its minimum (11.52 ± 0.72 μmol O₂ g ⁻ ¹ FW h ⁻ ¹) and differed significantly from all other treatment groups (*P* < 0.05). Compared with the IC group, the Rd values in the 1U2G, 1U4G, and 1U6G groups were 37.50%, 30.21%, and 23.96% higher, respectively. Under high light intensity (Fig 3B), R_d_ of both *G. lemaneiformis* and *U. prolifera* showed significant differences between the IC group and the 1U4G and 1U6G groups (*P* < 0.05). R_d_ peaked in the IC group for both species under HL: 13.56 ± 0.91 μmol O₂ g ⁻ ¹ FW h ⁻ ¹ for *G. lemaneiformis* and 16.92 ± 0.95 μmol O₂ g ⁻ ¹ FW h ⁻ ¹ for *U. prolifera*. With increasing *G. lemaneiformis* biomass density, R_d_ decreased slightly for both algae. R_d_ of *G. lemaneiformis* in the 1U4G and 1U6G groups decreased significantly by 27.43% and 25.66%, respectively, compared to IC. R_d_ of *U. prolifera* in the 1U4G and 1U6G groups decreased by 14.89% and 14.18%, respectively, compared to IC. Under the same culture conditions, the R_d_ of *U. prolifera* was significantly lower than that of *G. lemaneiformis* only in the low-light IC group (*P* < 0.05). R_d_ of *U. prolifera* was significantly higher than that of *G. lemaneiformis* (*P* < 0.05) in other treatment groups.

Two-way ANOVA (S2 Table in S1 File) indicated that the interaction between light intensity and biomass density did not significantly affect the net photosynthetic rate (P_n_) of *G. lemaneiformis* and *U. prolifera* (*P* > 0.05), but changes in light intensity and biomass density individually had significant effects on P_n_ (*P* < 0.05). As shown in Fig 3C-D, under the same light intensity, P_n_ of both algae gradually decreased with increasing biomass density of *G. lemaneiformis*. This trend paralleled the changes in relative growth rate, suggesting that elevated *G. lemaneiformis* biomass density suppressed P_n_ in both species. Under low light conditions, P_n_ of *G. lemaneiformis* and *U. prolifera* in monoculture were 18.60 ± 1.50 μmol O₂ g ⁻ ¹ FW h ⁻ ¹ and 23.76 ± 1.30 μmol O₂ g ⁻ ¹ FW h ⁻ ¹, respectively. For both species, no significant difference in P_n_ was observed between the IC and 1U2G groups. Compared with the IC group, the P_n_ of *G. lemaneiformis* decreased by 18.06% and 32.26% in the 1U4G and 1U6G groups, respectively, while that of *U. prolifera* decreased by 33.33% and 35.86% (Fig 3C). Under high light intensity, P_n_ of *G. lemaneiformis* showed no significant difference between the 1U2G group and the IC or 1U4G groups (*P* > 0.05), but a significant difference existed between IC and 1U4G groups (*P* < 0.05). P_n_ of *U. prolifera* showed no significant difference between the 1U2G and 1U4G groups (*P* > 0.05), but significant differences existed between IC and 1U6G groups (*P* < 0.05). P_n_ of both species peaked in the IC group under HL: 21.72 ± 1.62 μmol O₂ g ⁻ ¹ FW h ⁻ ¹ for *G. lemaneiformis* and 25.92 ± 1.90 μmol O₂ g ⁻ ¹ FW h ⁻ ¹ for *U. prolifera*. Compared to IC, P_n_ of *G. lemaneiformis* decreased by 7.73%, 23.20%, and 44.20%, and P_n_ of *U. prolifera* decreased by 12.96%, 24.54%, and 38.89% in the respective co-culture groups (Fig 3D).

### Chlorophyll fluorescence parameters

**Fig 4:**
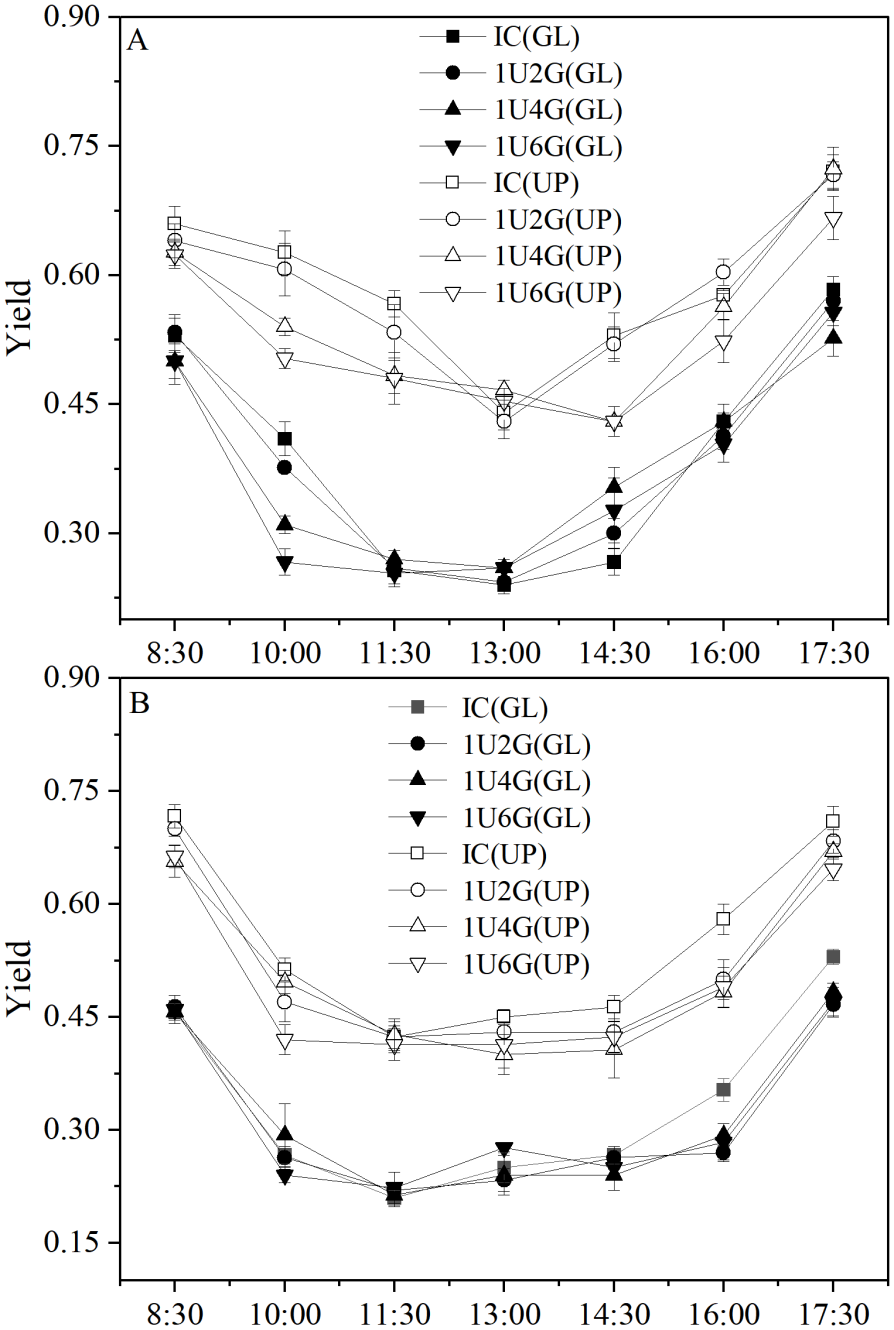
Diurnal variation of fluorescence quantum yield (Yield) for *G. lemaneiformis* and *U. prolifera* under low light (LL; A) and high light (HL; B) intensities at different culture densities. IC: Individual culture (monoculture); 1U2G, 1U4G, and 1U6G: Co-culture with *G. lemaneiformis* to *U. prolifera* biomass density ratios of 1:2, 1:4, and 1:6, respectively. Different lowercase letters indicate significant differences among *G. lemaneiformis* treatments at the same light intensity level across different culture densities. Different uppercase letters indicate significant differences among *U. prolifera* treatments at the same light intensity level across different culture densities. * indicates a significant difference between *G. lemaneiformis* and *U. prolifera* at the same culture density.

The diurnal variation of fluorescence quantum yield (Yield) for *G. lemaneiformis* and *U. prolifera* in various treatment groups is shown in Fig 4. Under low light intensity, the Yield of *G. lemaneiformis* at 8:00 AM was 0.53 ± 0.02, 0.53 ± 0.02, 0.50 ± 0.03, and 0.50 ± 0.02 for IC, 1U2G, 1U4G, and 1U6G groups, respectively. Yield decreased gradually from 8:30 AM to 1:00 PM, then showed an upward trend, peaking at 5:30 PM at 0.58 ± 0.02, 0.57 ± 0.01, 0.53 ± 0.02, and 0.56 ± 0.02 for the respective groups. For *U. prolifera* under LL, Yield at 8:00 AM was 0.66 ± 0.02, 0.64 ± 0.02, 0.63 ± 0.02, and 0.62 ± 0.02 for IC, 1U2G, 1U4G, and 1U6G groups. Yield in IC and 1U2G groups gradually decreased to a minimum from 8:30 AM to 1:00 PM, then increased. In 1U4G and 1U6G groups, Yield decreased to a minimum from 8:30 AM to 2:30 PM, then increased, also peaking at 5:30 PM at 0.72 ± 0.02, 0.72 ± 0.02, 0.72 ± 0.03, and 0.67 ± 0.03 for the respective groups (Fig 4A). Under high light intensity, Yield of *G. lemaneiformis* at 8:00 AM was 0.46 ± 0.01, 0.46 ± 0.02, 0.46 ± 0.02, and 0.46 ± 0.01 for IC, 1U2G, 1U4G, and 1U6G groups. Yield in IC, 1U2G, and 1U4G groups decreased to a minimum from 8:30 AM to 11:30 AM, then gradually increased. However, in the 1U6G group, Yield decreased gradually from 8:30 AM to 11:30 AM, increased from 11:30 AM to 1:00 PM, slightly decreased from 1:00 PM to 2:30 PM, then showed an upward trend, peaking at 5:30 PM at 0.53 ± 0.01, 0.47 ± 0.02, 0.48 ± 0.01, and 0.47 ± 0.02 for the respective groups. For *U. prolifera* under HL, Yield at 8:00 AM was 0.72 ± 0.02, 0.70 ± 0.01, 0.66 ± 0.02, and 0.66 ± 0.02 for IC, 1U2G, 1U4G, and 1U6G groups. Yield in all groups gradually decreased from 8:30 AM to 1:00 PM, then showed an upward trend, peaking at 5:30 PM at 0.71 ± 0.02, 0.68 ± 0.02, 0.67 ± 0.01, and 0.65 ± 0.02 for the respective groups (Fig 4B). Furthermore, under the same culture conditions, the Yield of *U. prolifera* was significantly higher than that of *G. lemaneiformis*.

**Fig 5:**
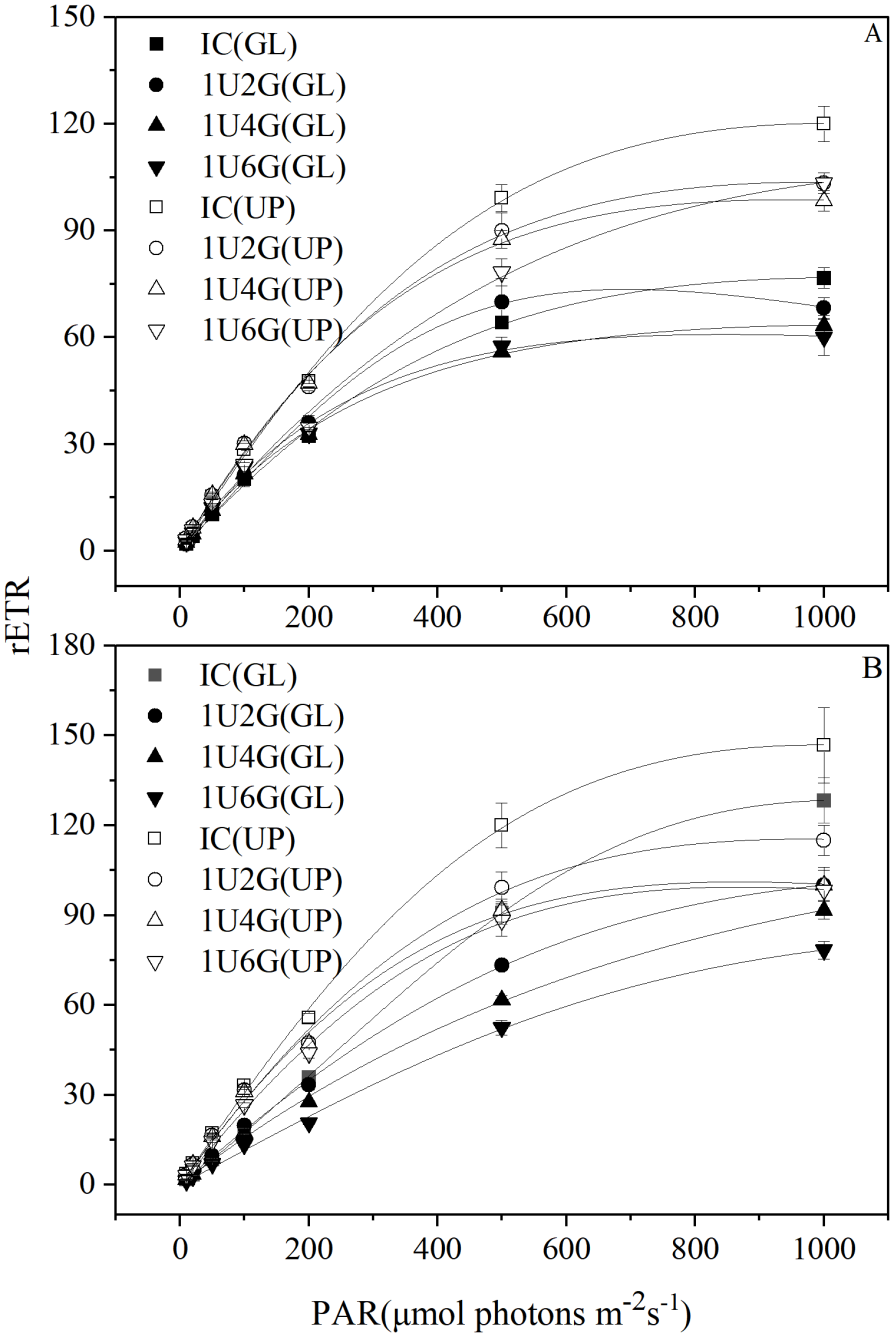
Relative Electron Transport Rate (rETR) for *G. lemaneiformis* and *U. prolifera* under low light (LL; A) and high light (HL; B) intensities at different culture densities. IC: Individual culture (monoculture); 1U2G, 1U4G, and 1U6G: Co-culture with *G. lemaneiformis* to *U. prolifera* biomass density ratios of 1:2, 1:4, and 1:6, respectively. Different lowercase letters indicate significant differences among *G. lemaneiformis* treatments at the same light intensity level across different culture densities. Different uppercase letters indicate significant differences among *U. prolifera* treatments at the same light intensity level across different culture densities. * indicates a significant difference between *G. lemaneiformis* and *U. prolifera* at the same culture density.

The rapid light response curves (RLCs) for *G. lemaneiformis* and *U. prolifera* in various treatment groups are shown in Fig 5. Under low light intensity, the relative electron transport rate (rETR) of *G. lemaneiformis* in monoculture and the 1U4G group, and *U. prolifera* in all groups, showed a gradual upward trend with increasing light intensity. In contrast, rETR of *G. lemaneiformis* in the 1U2G and 1U6G groups initially increased and then decreased. Under high light intensity, rETR of *G. lemaneiformis* in all groups, and *U. prolifera* in the IC and 1U2G groups, showed a stable upward trend with increasing light intensity. However, rETR of *U. prolifera* in the 1U4G and 1U6G groups increased initially and then decreased with further elevation of light intensity.

According to two-way ANOVA (S3 Table in S1 File), light intensity and biomass density each exerted a significant effect on the rETRₘₐₓ, α, and Iₖ of both *G. lemaneiformis* and *U. prolifera* (*P* < 0.05). Their interaction also significantly affected these three photosynthetic parameters (*P* < 0.05) (Table 1). Under low light conditions, with increasing culture biomass density, rETRₘₐₓ and Iₖ of *G. lemaneiformis* showed a gradual decreasing trend. rETRₘₐₓ in the 1U4G and 1U6G groups decreased significantly by 16.92% and 20.24% compared to IC (*P* < 0.05). Iₖ in the 1U6G group decreased significantly by 37.26% compared to IC (*P* < 0.05). α showed a gradually increasing trend. For *U. prolifera* under LL, rETRₘₐₓ and Iₖ first decreased and then increased. rETRₘₐₓ in the 1U2G and 1U4G groups decreased significantly by 12.70% and 17.74% compared to IC (*P* < 0.05). α first increased and then decreased. Under high light intensity, rETRₘₐₓ and α of *G. lemaneiformis* remained relatively stable until biomass density increased to the 1U6G group, where they decreased significantly by 33.52% and 29.29%, respectively, compared to IC (*P* < 0.05). Iₖ showed a trend of first decreasing and then rising. For *U. prolifera* under HL, rETRₘₐₓ and α showed a gradually decreasing trend. rETRₘₐₓ in the 1U2G, 1U4G, and 1U6G groups decreased significantly by 20.79%, 30.97%, and 31.27%, respectively, compared to IC. Iₖ remained relatively stable, with no significant differences (*P* > 0.05).

**Table 1:** Chlorophyll fluorescence parameters derived from rapid light response curves for *G. lemaneiformis* and *U. prolifera* under different culture conditions. IC stands for individually cultured *G. lemaneiformis* (GL) and *U. prolifera* (UP), 1U2G, 1U4G, and 1U6G: Co-culture with *G. lemaneiformis* to *U. prolifera* biomass density ratios of 1:2, 1:4, and 1:6, respectively. Different lowercase letters indicate significant differences among *G. lemaneiformis* treatments at the same light intensity level across different culture densities. Different uppercase letters indicate significant differences among *U. prolifera* treatments at the same light intensity level across different culture densities. * indicates a significant difference between *G. lemaneiformis* and *U. prolifera* at the same culture density. The data are the mean ± the standard deviation (n = 3).

	Treatment	rETR_max_	I_k_	α
low light	IC(GL)	76.90 ± 3.02^c^	384.90 ± 37.88^b^	0.20 ± 0.02^a^
	1U2G(GL)	73.74 ± 6.21^bc^	379.18 ± 85.42^b^	0.20 ± 0.03^a^
	1U4G(GL)	63.89 ± 3.19^ab^	261.87 ± 11.08^ab^	0.24 ± 0.01^ab^
	1U6G(GL)	61.33 ± 3.69^a^	241.49 ± 15.30^a^	0.25 ± 0.01^b^
	IC(UP)	120.50 ± 5.40^B*^	433.13 ± 5.89^B^	0.28 ± 0.01^B*^
	1U2G(UP)	105.19 ± 4.00^A*^	348.66 ± 10.61^A^	0.30 ± 0.01^B*^
	1U4G(UP)	99.12 ± 2.63^A*^	320.13 ± 16.93^A*^	0.31 ± 0.02^B*^
	1U6G(UP)	108.24 ± 134.62^AB*^	487.25 ± 16.38^C*^	0.23 ± 0.02^A*^
High light	IC(GL)	129.15 ± 7.80^b^	782.14 ± 32.82^b^	0.17 ± 0.01^b^
	1U2G(GL)	103.44 ± 7.96^ab^	550.26 ± 71.04^a^	0.19 ± 0.02^b^
	1U4G(GL)	117.38 ± 19.31^b^	697.81 ± 102.81^ab^	0.17 ± 0.01^b^
	1U6G(GL)	85.86 ± 4.45^a^	738.65 ± 39.20^b^	0.12 ± 0.01^a^
	IC(UP)	146.92 ± 12.53^B^	469.11 ± 45.96^B*^	0.31 ± 0.01^A*^
	1U2G(UP)	116.38 ± 5.67^A^	392.49 ± 27.37^AB*^	0.30 ± 0.02^A*^
	1U4G(UP)	101.41 ± 4.94^A^	335.37 ± 35.65^A*^	0.30 ± 0.02^A*^
	1U6G(UP)	100.98 ± 4.95^A*^	387.49 ± 41.70^AB*^	0.26 ± 0.03^A*^

Notes: rETR_max_, maximum relative electron transfer rate; α, light utilization efficiency; I_k_, photosynthesis saturation light intensity point.

### Pigments content

**Fig 6:**
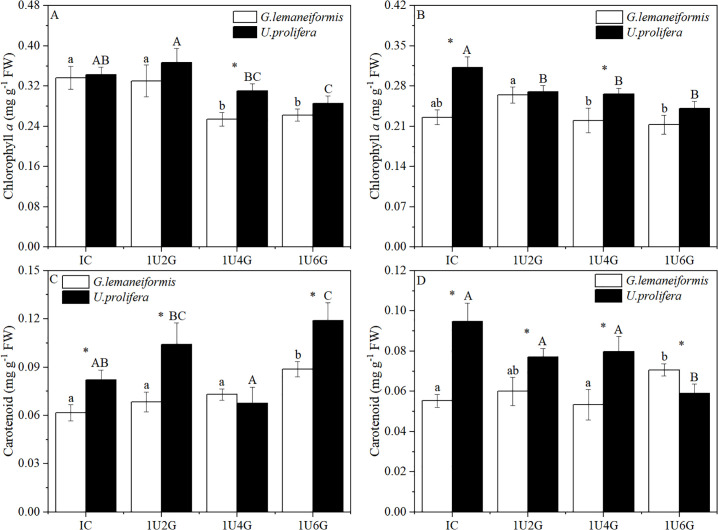
Chlorophyll *a* (Chl *a*; A, B) and carotenoid (Car.; C, D) content of *G. lemaneiformis* and *U. prolifera* under low light (LL; A, C) and high light (HL; B, D) intensities at different culture densities. IC: Individual culture (monoculture); 1U2G, 1U4G, and 1U6G: Co-culture with *G. lemaneiformis* to *U. prolifera* biomass density ratios of 1:2, 1:4, and 1:6, respectively. Different lowercase letters indicate significant differences among *G. lemaneiformis* treatments at the same light intensity level across different culture densities. Different uppercase letters indicate significant differences among *U. prolifera* treatments at the same light intensity level across different culture densities. * indicates a significant difference between *G. lemaneiformis* and *U. prolifera* at the same culture density.

Two-way ANOVA revealed that chlorophyll *a* (Chl *a*) and Carotenoid (Car.) content of the algae were significantly affected by light intensity and biomass density (*P* < 0.05), and their interaction also exerted a significant effect on Chl *a* and Car. content (*P* < 0.05) (S4 Table in S1 File). As shown in Fig 6A, under low light intensity, Chl *a* of *G. lemaneiformis* showed no significant difference between IC and 1U2G groups (*P* > 0.05), but significant differences compared to 1U4G and 1U6G groups (*P* < 0.05), decreasing significantly by 26.47% and 23.53% compared to IC. Chl *a* of *U. prolifera* in the 1U2G group was significantly different from that in the 1U4G and 1U6G groups (*P* < 0.05), decreasing by 16.22% and 21.62%, respectively, compared to the 1U2G group. Under high light intensity (Fig 6B), Chl *a* of *G. lemaneiformis* showed no significant difference among IC, 1U2G, 1U4G, and 1U6G groups (*P* > 0.05). Chl *a* in the 1U4G and 1U6G groups decreased significantly by 15.38% and 19.23% compared to the 1U2G group (*P* < 0.05). Chl *a* of *U. prolifera* in the 1U2G, 1U4G, and 1U6G groups decreased significantly by 12.90%, 12.90%, and 22.58%, respectively, compared to IC (*P* < 0.05). Under both LL and HL conditions, Chl *a* of *U. prolifera* was significantly higher than that of *G. lemaneiformis* only in the 1U4G group, by 19.35% and 18.52%, respectively (*P* < 0.05).

Under low light intensity, Car. content of *G. lemaneiformis* showed no significant difference among IC, 1U2G, and 1U4G groups (*P* > 0.05), but was significantly lower in these groups compared to the 1U6G group, with decreases of 33.33%, 22.22%, and 22.22% respectively (*P* < 0.05). Car. content of *U. prolifera* showed no significant difference between IC and 1U2G or 1U4G groups (*P* > 0.05), but IC and 1U4G groups were significantly lower than 1U6G by 33.33% and 41.67%, respectively (Fig 6C). Under high light intensity, Car. content of *G. lemaneiformis* showed no significant difference among IC, 1U2G, and 1U4G groups (*P* > 0.05). IC and 1U4G groups were significantly lower than 1U6G by 14.29% and 28.57% (*P* < 0.05). Car. content of *U. prolifera* in IC, 1U2G, and 1U4G groups was significantly higher than in 1U6G by 50.00%, 33.33%, and 33.33% (*P* < 0.05). Under low light, Car. content of *U. prolifera* was significantly higher than that of *G. lemaneiformis* in IC, 1U2G, and 1U6G groups by 33.33%, 42.86%, and 33.33% (*P* < 0.05) (Fig 6C). Under high light, Car. content of *U. prolifera* was significantly higher than that of *G. lemaneiformis* in IC, 1U2G, and 1U4G groups by 69.64%, 33.33%, and 60.00% (*P* < 0.05), but significantly lower by 14.29% in the 1U6G group (*P* < 0.05) (Fig 6D).

**Fig 7:**
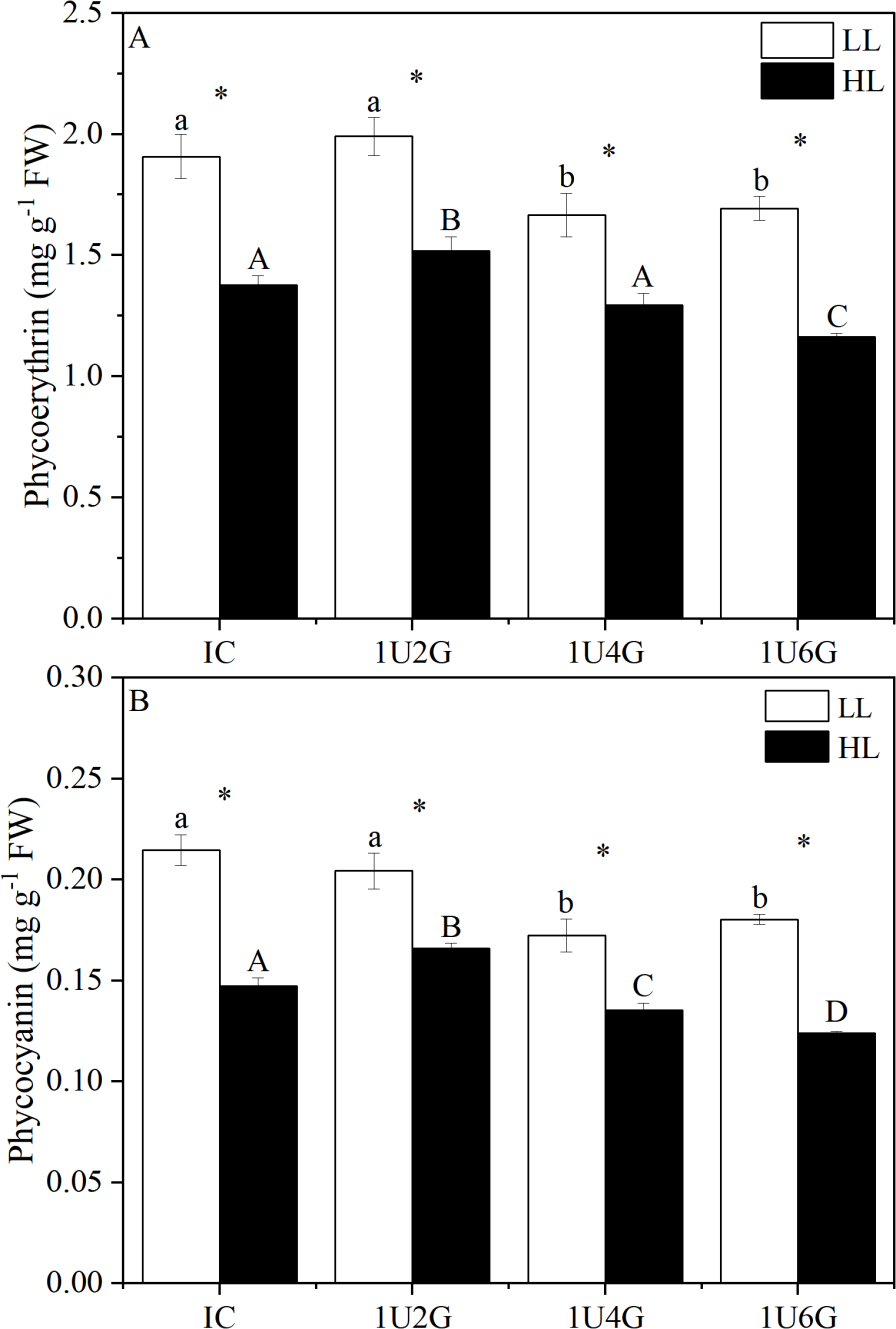
Phycoerythrin (PE; A) and phycocyanin (PC; B) content of *G. lemaneiformis* under low light (LL) and high light (HL) intensities at different culture densities. IC: Individual culture (monoculture); 1U2G, 1U4G, and 1U6G: Co-culture with *G. lemaneiformis* to *U. prolifera* biomass density ratios of 1:2, 1:4, and 1:6, respectively. Different lowercase letters indicate significant differences among *G. lemaneiformis* treatments at the same density under HL. Different uppercase letters indicate significant differences among *G. lemaneiformis* treatments at the same density under LL. * indicates a significant difference between LL and HL treatments for the same culture density.

The phycoerythrin (PE) and phycocyanin (PC) content of *G. lemaneiformis* were significantly affected by light intensity and biomass density (*P* < 0.05). The interaction between light intensity and biomass density significantly affected PC content (*P* < 0.05), but not PE content (*P* > 0.05) (S5 Table in S1 File). Under low light intensity (Fig 7A-B), no significant differences in PE or PC content were detected between the IC and 1U2G groups (P > 0.05) or between the 1U4G and 1U6G groups (P > 0.05). Under high light intensity, PE content in the 1U2G and 1U6G groups differed significantly from IC (*P* < 0.05). PE and PC content in the 1U2G group increased significantly by 10.14% and 13.33% compared to IC, while in the 1U6G group, PE and PC content decreased significantly by 15.94% and 20.00% compared to IC. PE and PC content of *G. lemaneiformis* were significantly higher under low light intensity than under high light intensity.

### Soluble protein and soluble carbohydrate content

**Fig 8:**
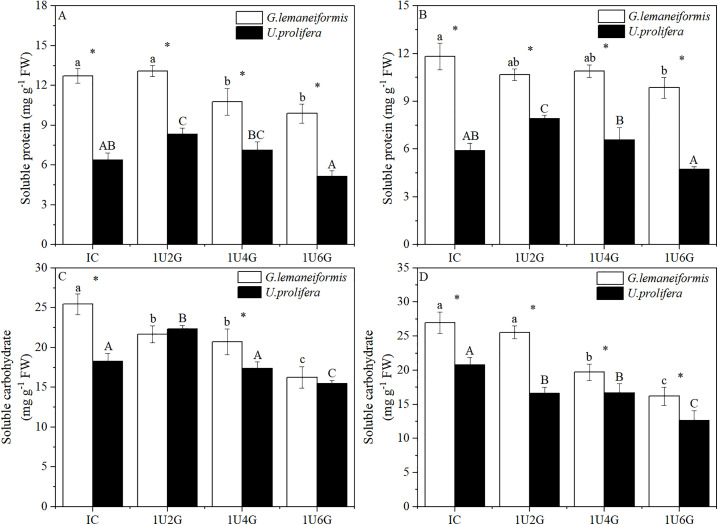
Soluble protein (SP; A, B) and soluble carbohydrate (SC; C, D) content of *G. lemaneiformis* and *U. prolifera* under low light (LL; A, C) and high light (HL; B, D) intensities at different culture densities. IC: Individual culture (monoculture); 1U2G, 1U4G, and 1U6G: Co-culture with *G. lemaneiformis* to *U. prolifera* biomass density ratios of 1:2, 1:4, and 1:6, respectively. Different lowercase letters indicate significant differences among *G. lemaneiformis* treatments at the same light intensity level across different culture densities. Different uppercase letters indicate significant differences among *U. prolifera* treatments at the same light intensity level across different culture densities. * indicates a significant difference between *G. lemaneiformis* and *U. prolifera* at the same culture density.

Changes in soluble protein (SP) and soluble carbohydrate (SC) content of *G. lemaneiformis* and *U. prolifera* under different treatments are shown in Fig 8. SP content was significantly affected by light intensity and biomass density (*P* < 0.05), but their interaction did not significantly affect SP (*P* > 0.05). Light intensity did not significantly affect SC content (*P* > 0.05), while biomass density significantly affected SC content of both species (*P* < 0.05). The interaction between light intensity and biomass density significantly affected both SP and SC content (*P* < 0.05) (S6 Table in S1 File). As shown in Fig 8A, under low light intensity, SP content of *G. lemaneiformis* showed no significant difference between IC and 1U2G groups (*P* > 0.05), but decreased significantly by 15.33% and 22.33% in the 1U4G and 1U6G groups compared to IC (*P* < 0.05). SP content of *U. prolifera* in the 1U2G group increased significantly by 30.61% compared to IC (*P* < 0.05). As shown in Fig 8B, under high light intensity, SP content of *G. lemaneiformis* showed no significant difference among IC, 1U2G, and 1U4G groups (*P* > 0.05), but decreased significantly by 16.58% in the 1U6G group compared to IC (*P* < 0.05). SP content of *U. prolifera* first increased and then decreased with increasing biomass density (Fig 8A-B); under high light intensity, SP in the 1U2G group increased significantly by 34.24% compared to IC (*P* < 0.05), while SP in the 1U4G and 1U6G groups showed no significant difference from IC (*P* > 0.05). Under the same treatment conditions, the SP content of *G. lemaneiformis* was significantly higher than that of *U. prolifera* (*P* < 0.05).

As shown in Fig 8C, under low light intensity, SC content of *G. lemaneiformis* reached its highest value in the IC group (25.43 ± 1.29 mg g ⁻ ¹ FW). SC content in the 1U2G, 1U4G, and 1U6G groups decreased significantly by 14.83%, 18.48%, and 36.06%, respectively, compared to IC (*P* < 0.05). SC content of *U. prolifera* in the 1U2G group increased significantly by 22.17% compared to IC (*P* < 0.05), while in the 1U6G group, it decreased significantly by 15.44% (*P* < 0.05). SC content of *G. lemaneiformis* was significantly higher than that of *U. prolifera* by 29.83% and 18.18% in the IC and 1U4G groups, respectively (*P* < 0.05) (Fig 8C-D). As shown in Fig 8D, under high light intensity, SC content of both *G. lemaneiformis* and *U. prolifera* decreased significantly with increasing biomass density. SC of *G. lemaneiformis* in the 1U4G and 1U6G groups decreased significantly by 26.87% and 39.90% compared to IC (*P* < 0.05). SC of *U. prolifera* in the 1U2G, 1U4G, and 1U6G groups decreased significantly by 20.10%, 19.66%, and 39.04%, respectively, compared to IC (*P* < 0.05). In all HL treatment groups, the SC content of *G. lemaneiformis* was significantly higher than that of *U. prolifera* (*P* < 0.05).

## Discussion

The competitive relationships among macroalgae are a critical issue in coastal ecological regulation and algal bloom bioremediation. Their interspecific interactions directly shape community structure, influence nutrient cycling, light utilization efficiency, and niche partitioning, thereby determining the potential and scale of algal blooms. By analyzing how key environmental factors—such as light, temperature, nutrients, and water flow—regulate competitive dynamics, a theoretical foundation can be established for developing niche-competition-based “using algae to control algae” biocontrol technologies. In this study, the inhibitory effect of *G. lemaneiformis* on the growth of *U. prolifera* was investigated by regulating light intensity and biomass density ratios.

This study found that the relative growth rate and photosynthetic efficiency of *U. prolifera* significantly decreased with increasing *G. lemaneiformis* proportion under co-culture conditions, which can be attributed to the accumulation of allelopathic inhibitory compounds. Marine macroalgae produce various secondary metabolites, such as terpenes, sterols, polyphenols, and acetogenins, to protect themselves from herbivores or other phototrophs. Generally, allelopathy may involve interactions with proteins, inhibition of alkaline phosphatase, disruption of the electron transport chain, membrane damage, and oxidative damage caused by polyphenol auto-oxidation [27]. Previous studies have confirmed that *G. lemaneiformis* can inhibit *Ulva* growth by releasing specific allelochemicals [14]. Culture filtrate of *G. lemaneiformis* significantly reduces biomass accumulation and PSII effective quantum yield (Yield) of *U. prolifera*, with stronger inhibition at higher filtrate concentrations. In this study, the carotenoid content of *U. prolifera* was lower than that of *G. lemaneiformis* (by 14.29%) only in the high-light 1U6G group. Carotenoids are key pigments defending against photo-oxidative damage. Their reduction makes *U. prolifera* more susceptible to light stress under high light. Further studies suggest this may be related to hydrogen peroxide (H₂O₂) and halogenated hydrocarbons released by *G. lemaneiformis*, which can disrupt photosynthetic pigment synthesis and photosystem stability in *U. prolifera*, leading to increased susceptibility to photodamage under high light [12]. The more significant decrease in photosynthetic parameters of *U. prolifera* in high-biomass density *G. lemaneiformis* groups under high light in this study may be related to this mechanism.

Interactions among macroalgae involve complex interspecific competition mediated by both direct and indirect mechanisms. *Neopyropia yezoensis* can inhibit gamete germination in *U. prolifera* through allelopathic effects exerted by its dried powder, fresh thalli, and culture filtrates [28]. Conversely, when co-cultured, *U. prolifera* significantly suppresses the growth and net photosynthetic rate of *N. yezoensis*. Elevated CO_2_ concentrations reduce the growth rate of *N. yezoensis* while simultaneously enhancing its resistance to allelopathic stress from *U. prolifera* [29]. The cultivation of *G. lemaneiformis* can enhance plankton biodiversity through allelopathy by suppressing dominant plankton species [30]. However, in direct interactions between *G. lemaneiformis* and *U. prolifera*, the presence of *U. prolifera* inhibits the growth of *G. lemaneiformis*, whereas *G. lemaneiformis* promotes the growth of *U. prolifera*—though this promotive effect can be offset by higher biomass densities [19]. These findings reveal the nuanced roles of allelopathy, environmental factors such as CO₂, and density-dependent effects in shaping macroalgal community dynamics.

Resource competition is a fundamental driver of macroalgal growth. As a typical opportunistic alga, the rapid growth of *U. prolifera* depends on sustained light and nutrient supply [5]. In this study, *G. lemaneiformis* potentially compressed the ecological space of *U. prolifera*, affecting localized light conditions and nutrient availability. The shading effect induced by *G. lemaneiformis* led to a reduction in chlorophyll *a* content and light energy utilization efficiency in *U. prolifera*, thereby inhibiting its growth. Studies show that while the nitrogen uptake rate of *U. prolifera* is higher than that of *G. lemaneiformis*, the latter possesses stronger nitrogen storage capacity, storing nitrogen in the form of phycoerythrin, which may allow it to maintain a growth advantage in low-nutrient environments [12]. Simultaneously, the efficient absorption of nitrogen and phosphorus by *G. lemaneiformis* further restricts nutrient acquisition by *U. prolifera*, manifested as inhibition of nitrate reductase (NR) activity and soluble protein (SP) synthesis in *U. prolifera* [31,32]. This aligns with previous conclusions that *Gracilariopsis spp.* significantly inhibits *Ulva spp.* growth through resource competition, with the effect intensifying with increasing biomass density [19,33,34].

Light is the driving source for photosynthesis in macroalgae and an important environmental factor affecting their productivity. Light intensity is also a major abiotic factor influencing algal growth and photosynthesis; both excessively high and low intensities affect photosynthesis and consequently normal growth [35,36]. It is noteworthy that in the Yellow Sea green tide outbreak areas, although floating *U. prolifera* aggregations are primarily concentrated in the well-lit surface layer, the high turbidity of nearshore waters causes light to attenuate rapidly with water depth. The low-light and high-light treatments established in this experiment were designed to simulate two representative light environments that algae may experience in nearshore settings: one being light-limited conditions influenced by factors such as water turbidity or internal shading, and the other representing higher light conditions closer to the surface or under clear weather conditions. The relative growth rate and P_n_ of both *G. lemaneiformis* and *U. prolifera* in monoculture were higher under high light intensity than under low light intensity, indicating that appropriately increased light intensity benefits algal growth and photosynthetic physiology. This result is consistent with studies on other large seaweeds such as *Undaria pinnatifida* [37], *Gracilaria lemaneiformis* [38], and *Gracilaria asiatica* [39]. Under high light, rETRₘₐₓ and Iₖ of *G. lemaneiformis* were significantly higher than under low light, consistent with the trends in relative growth rate and P_n_. A similar, though less pronounced, trend was observed for *U. prolifera*. Macroalgae regulate their pigment content, thallus structure, and morphology in response to long-term exposure to different light levels [40]. Enhanced pigment synthesis under low light requires more energy, leading to lower growth than under high light. However, under high light, Chl *a* and Car. content of both *G. lemaneiformis* and *U. prolifera* decreased compared to low light, and *G. lemaneiformis* downregulated the synthesis of PE and PC. This may be because high light inhibits photosynthetic pigment synthesis and promotes thallus growth, a pattern described as exhibiting “pigment economy” [41,42]. Therefore, under high-light conditions, the shading effect of *G. lemaneiformis* itself and its potential disruption to the photosystem of *U. prolifera* are amplified. This, to some extent, simulates the light competition effect that high-biomass *G. lemaneiformis* cultivation areas may exert on surface or subsurface *U. prolifera*, although it differs from the bloom scenario with unlimited light availability in open-sea surface waters.

Biomass density is a key factor regulating algal photosynthesis and growth, directly affecting critical environmental parameters such as available light intensity, nutrient concentration, dissolved oxygen, and inorganic carbon content [43]. When algae share the same ecological niche, this biomass density effect inevitably triggers interspecific resource competition. In this study, as biomass density increased, the relative growth rate and P_n_ of both *G. lemaneiformis* and *U. prolifera* decreased and were significantly lower than in monoculture controls (*P* < 0.05). This inhibition may stem from light attenuation and nutrient limitation caused by increased biomass density. Notably, increased *G. lemaneiformis* biomass exhibited a significant inhibitory effect on *U. prolifera* growth (*P* < 0.05), and the inhibition intensity strengthened with increasing *G. lemaneiformis* biomass density. This result aligns with reports of *G. lemaneiformis* competitively excluding dinoflagellates [44,45]. Previous studies indicate that nutrient levels in high-biomass density *G. lemaneiformis* cultivation are lower than in low-biomass density cultivation. Increased consumption may lead to inorganic carbon and nutrient shortages in high-biomass density *G. lemaneiformis* cultures, thereby inhibiting photosynthesis [46]. Similarly, due to the presence of *U. prolifera*, the growth rate of *G. lemaneiformis* also decreased with increasing biomass density, though less significantly than for *U. prolifera*, indicating competitive interaction. Previous studies have shown that when stocking biomass density increases, algal nitrate reductase (NR) activity significantly decreases. This suggests that high stocking biomass density reduces nitrogen metabolic capacity, which potentially explains the decrease in relative growth rate (RGR) observed in high-biomass density seaweed cultures [46]. When the two species were co-cultured, *G. lemaneiformis* exhibited a competitive advantage.

Previous studies have confirmed the effects of light intensity and biomass density on the growth and physiological performance of *U. prolifera* and *G. lemaneiformis* individually, but knowledge of their interaction is limited. With increasing co-culture biomass density, the relative growth rate, net photosynthetic rate, and chlorophyll fluorescence parameters of both *G. lemaneiformis* and *U. prolifera* significantly decreased compared to monoculture [46]. The inhibitory effect of *G. lemaneiformis* on *U. prolifera* under low light was weaker than under high light, possibly related to differences in their light adaptation strategies. Macroalgal growth is negatively correlated with stocking biomass density. Under low light, the RGR of *U. prolifera* decreased by 36.92% at high stocking biomass density, while under high light, it decreased by 45.10%. The extent to which increased culture biomass density inhibited the growth rates of both *U. prolifera* and *G. lemaneiformis* was itself mediated by light intensity [47]. However, under low light, the decreasing trend was less pronounced, possibly because *G. lemaneiformis* growth was lower, making the biomass density effect less significant. Under high light, the growth rate decrease of high-biomass density *G. lemaneiformis* was significant, and soluble protein content changed little, but the decrease was less pronounced than for *U. prolifera*. This indicates that high light intensity and high *G. lemaneiformis* culture biomass density significantly inhibited the growth of *U. prolifera*.

This study systematically analyzed the interactive effects of light intensity and biomass density on the competitive relationship between *G. lemaneiformis* and *U. prolifera*, revealing that *G. lemaneiformis* suppresses the growth of *U. prolifera* through shading effects and potential allelopathic interactions. These findings deepen the understanding of interspecific competition mechanisms among macroalgae and provide experimental support for near-shore ecological regulation based on the “using algae to control algae” concept. Additionally, they offer scientific guidance for optimizing the cultivation layout of *G. lemaneiformis* in specific nearshore waters (e.g., eutrophic bays and aquaculture areas) during green tide outbreaks.

## Conclusions

In recent years, the recurrent and persistent outbreaks of *U. prolifera*-induced green tides in the Yellow Sea have posed severe threats to coastal ecosystem health and coastal-related industries. Although various mitigation strategies have been deployed to address this issue, they often incur high economic costs, substantial resource consumption, and potential environmental risks. This in situ study, conducted in a key *U. prolifera* bloom source area, demonstrates that the competitive outcome between *G. lemaneiformis* and *U. prolifera* is significantly regulated by the interactive effects of light intensity and biomass density. Specifically, increasing *G. lemaneiformis* biomass density reduces the relative growth rate (RGR) of *U. prolifera*, and this inhibitory effect is particularly pronounced under high-light conditions. The inhibitory effect is primarily achieved through mechanisms such as shading effects and potential allelopathic interactions. Notably a biomass density ratio of 1:4–1:6 (*U. prolifera*: *G. lemaneiformis*) was identified as an optimal ratio range that effectively suppresses *U. prolifera* growth while sustaining the cultivation of *G. lemaneiformis*. These findings confirm the feasibility of developing interspecific competition-based biological control strategies for green tides, while providing empirical data to support the optimizing of *G. lemaneiformis* cultivation and the advancement of green tide bioremediation.

## Supporting information

S1 File The data in paper analysis. (PDF)
