# Supplementary material for: Effects of co-cultivation with Gracilariopsis lemaneiformis on the explosive growth and photosynthesis of Ulva prolifera
Source: PLoS One. 2026 Mar 10;21(3):e0344202. doi: 10.1371/journal.pone.0344202 (PMC12974846; doi:10.1371/journal.pone.0344202)
Supplement: S1 File — (PDF) [file pone.0344202.s001.pdf]

S1 Table. Two-way analysis of variance for the effects of light intensity and biomass density on RGR of *Gracilariopsis lemaneiformis and Ulva prolifera.*

Treatments	df	F	Sig.
RGR			
Light intensity	1	29.533	<0.001
Biomass density	3	169.218	<0.001
Algal species	1	277.986	<0.001
Light intensity*Biomass density	3	15.982	<0.001
Light intensity*Algal species	1	0.427	0.518
Biomass density*Algal species	3	4.907	0.006
Light intensity*Biomass density*Algal species	3	2.264	0.100

Notes: light intensity*biomass density represents the interactive effects of light intensity and biomass density; Light intensity*algal species represents the interactive effects of light intensity and algal species; biomass density*algal species represents the interactive effects of biomass density and algal species; RGR, relative growth rate; df, degree of freedom; F, F statistic value; Sig., p value.

S2 Table. A two-way analysis of variance was performed on the effects of light intensity and biomass density on the R_d and P_n of *Gracilariopsis lemaneiformis* and *Ulva prolifera*.

Treatments	df	F	Sig.
R_d			
Light intensity	1	0.068	0.796
Biomass density	3	11.681	<0.001
Algal species	1	98.864	<0.001
Light intensity*Biomass density	3	5.538	0.004
Light intensity*Algal species	1	20.512	<0.001
Biomass density*Algal species	3	12.512	<0.001
Light intensity*Biomass density*Algal species	3	6.832	0.001
P_n			
Light intensity	1	24.48	<0.001
Biomass density	3	103.829	<0.001
Algal species	1	72.533	<0.001
Light intensity*Biomass density	3	2.678	0.064
Light intensity*Algal species	1	0.237	0.630
Biomass density*Algal species	3	2.651	0.065
Light intensity*Biomass density*Algal species	3	1.171	0.336

Notes: light intensity*biomass density represents the interactive effects of light intensity and biomass density; Light intensity*algal species represents the interactive effects of light intensity and algal species; biomass density*algal species represents the interactive effects of biomass density and algal species; P_n, net photosynthetic rate; R_d, dark respiration rate;df, degree of freedom; *F*, F statistic value; Sig., *p* value.

S3 Table. A two-factor analysis of variance on the effects of light intensity and biomass density on the chlorophyll fluorescence parameters of *Gracilariopsis lemaneiformis* and *Ulva prolifera*.

Treatments	df	F	Sig.
rETRmax			
Light intensity	1	118.447	<0.001
Biomass density	3	32.245	<0.001
Algal species	1	116.773	<0.001
Light intensity*Biomass density	3	9.274	<0.001
Light intensity*Algal species	1	54.699	<0.001
Biomass density*Algal species	3	5.554	0.003
Light intensity*Biomass density*Algal species	3	2.575	0.071
Ik			
Light intensity	1	198.288	<0.001
Biomass density	3	14.918	<0.001
Algal species	1	65.877	<0.001
Light intensity*Biomass density	3	4.168	0.013
Light intensity*Algal species	1	200.792	<0.001
Biomass density*Algal species	3	2.733	0.060
Light intensity*Biomass density*Algal species	3	13.324	<0.001
α			
Light intensity	1	24.598	<0.001
Biomass density	3	12.709	<0.001
Algal species	1	361.397	<0.001
Light intensity*Biomass density	3	6.135	0.002
Light intensity*Algal species	1	63.796	<0.001
Biomass density*Algal species	3	5.803	0.003
Light intensity*Biomass density*Algal species	3	12.144	<0.001

Notes: light intensity*biomass density represents the interactive effects of light

intensity and biomass density; Light intensity*algal species represents the interactive effects of light intensity and algal species; Biomass density*algal species represents the interactive effects of biomass density and algal species; $rETR_{max}$, maximum relative electron transfer rate; I_k, saturated light intensity; α, light energy utilization efficiency;df, degree of freedom; *F*, F statistic value; Sig., *p* value.

S4 Table. A two-factor analysis of variance on the effects of light intensity and biomass density on the content of chlorophyll *a* and caroteniod in *Gracilariopsis lemaneiformis* and *Ulva prolifera*.

Treatments	df	F	Sig.
Chl *a*			
Light intensity	1	131.517	<0.001
Biomass density	3	31.358	<0.001
Algal species	1	48.46	<0.001
Light intensity*Biomass density	3	3.562	0.025
Light intensity*Algal species	1	1.146	0.292
Biomass density*Algal species	3	2.105	0.119
Light intensity*Biomass density*Algal species	3	5.449	0.004
Car.			
Light intensity	1	46.189	<0.001
Biomass density	3	10.143	<0.001
Algal species	1	81.162	<0.001
Light intensity*Biomass density	3	19.411	<0.001
Light intensity*Algal species	1	0.351	0.558
Biomass density*Algal species	3	6.311	0.002
Light intensity*Biomass density*Algal species	3	16.165	<0.001

Notes: light intensity*biomass density represents the interactive effects of light intensity and biomass density; Light intensity*algal species represents the interactive effects of light intensity and algal species; biomass density*algal species represents the interactive effects of biomass density and algal species; Chl *a*, chlorophyll *a*; Car., carotenoids; df, degree of freedom; F, *F* statistic value; Sig., *p* value.

S5 Table. A two-factor variance analysis of the effects of light intensity and biomass density on the content of phycoerythrin and phycocyanin in *Gracilariopsis lemaneiformis*.

Treatments	df	F	Sig.
PE			
Light intensity	1	335.284	<0.001
Biomass density	3	32.939	<0.001
Light intensity*Biomass density	3	2.084	0.143
PC			
Light intensity	1	480.984	<0.001
Biomass density	3	59.276	<0.001
Light intensity*Biomass density	3	10.452	<0.001

Notes: light intensity*biomass density represents the interactive effects of light intensity and biomass density; PE, phycoerythrin; PC, phycocyanin; df, degree of freedom; F, *F* statistic value; Sig., *p* value.

S6 Table. Results of a two-way analysis of variance on the effects of light intensity and biomass density on the content of soluble protein and soluble carbohydrates in *Gracilariopsis lemaneiformis* and *Ulva prolifera*.

Treatments	df	F	Sig.
SP			
Light intensity	1	13.937	0.001
Biomass density	3	41.732	<0.001
Algal species	1	781.449	<0.001
Light intensity*Biomass density	3	2.837	0.054
Light intensity*Algal species	1	1.071	0.308
Biomass density*Algal species	3	10.283	<0.001
Light intensity*Biomass density*Algal species	3	3.215	0.036
SC			
Light intensity	1	0.801	0.377
Biomass density	3	102.129	<0.001
Algal species	1	143.464	<0.001
Light intensity*Biomass density	3	5.254	0.005
Light intensity*Algal species	1	16.631	<0.001
Biomass density*Algal species	3	8.126	<0.001
Light intensity*Biomass density*Algal species	3	12.929	<0.001

Notes: light intensity*biomass density represents the interactive effects of light intensity and biomass density; Light intensity*algal species represents the interactive effects of light intensity and algal species; biomass density*algal species represents the interactive effects of biomass density and algal species; SP, soluble protein; SC, soluble carbohydrates; df, degree of freedom; *F*, F statistic value; Sig., *p* value.
